# Tiagabine Improves Hippocampal Long-Term Depression in Rat Pups Subjected to Prenatal Inflammation

**DOI:** 10.1371/journal.pone.0106302

**Published:** 2014-09-03

**Authors:** Aline Rideau Batista Novais, Nadine Crouzin, Mélanie Cavalier, Mathilde Boubal, Janique Guiramand, Catherine Cohen-Solal, Marie-Céleste de Jesus Ferreira, Gilles Cambonie, Michel Vignes, Gérard Barbanel

**Affiliations:** 1 Laboratory IBMM-UMR 5247 “Institut des Biomolécules Max Mousseron”, CNRS - Montpellier 1 University - Montpellier 2 University, Montpellier, France; 2 Neonatal Intensive Care Unit, Montpellier University Hospital, Montpellier, France; 3 Laboratory NICN-UMR7259 “Neurobiologie des Interactions Cellulaires et Neurophysiopathologie”, CNRS - Aix-Marseille University, Marseille, France; University of Kentucky, United States of America

## Abstract

Maternal inflammation during pregnancy is associated with the later development of cognitive and behavioral impairment in the offspring, reminiscent of the traits of schizophrenia or autism spectrum disorders. Hippocampal long-term potentiation and long-term depression of glutamatergic synapses are respectively involved in memory formation and consolidation. In male rats, maternal inflammation with lipopolysaccharide (LPS) led to a premature loss of long-term depression, occurring between 12 and 25 postnatal days instead of after the first postnatal month, and aberrant occurrence of long-term potentiation. We hypothesized this would be related to GABAergic system impairment. Sprague Dawley rats received either LPS or isotonic saline *ip* on gestational day 19. Male offspring's hippocampus was studied between 12 and 25 postnatal days. Morphological and functional analyses demonstrated that prenatal LPS triggered a deficit of hippocampal GABAergic interneurons, associated with presynaptic GABAergic transmission deficiency in male offspring. Increasing ambient GABA by impairing GABA reuptake with tiagabine did not interact with the low frequency-induced long-term depression in control animals but fully prevented its impairment in male offspring of LPS-challenged dams. Tiagabine furthermore prevented the aberrant occurrence of paired-pulse triggered long-term potentiation in these rats. Deficiency in GABA seems to be central to the dysregulation of synaptic plasticity observed in juvenile *in utero* LPS-challenged rats. Modulating GABAergic tone may be a possible therapeutic strategy at this developmental stage.

## Introduction

Epidemiological studies have repeatedly demonstrated that prenatal infection is associated with an increased risk for adverse neurological outcome and neuropsychiatric diseases, most particularly schizophrenia and autism spectrum disorders [1]–[3]. Experimental models have been developed to analyze the mechanisms involved in the pathological process, including neurodevelopmental aspects, and to provide etiological support for the design of new therapeutic strategies. In rodents, prenatal inflammation - for instance induced after injection of lipopolysaccharide (LPS) - indeed leads to cognitive deficits and behavioral changes (see for review [4]), structural abnormalities and neuronal network deficits [5]–[8].

Hippocampal glutamatergic long-term potentiation (LTP) and long-term depression (LTD) are considered as the electrophysiological substratum to memory formation [9] and consolidation [10]. Consistent with reduced learning abilities in the Morris watermaze, prenatally LPS-challenged males displayed a deficit in long-term potentiation (LTP) [11], [12]. These deficits, whether electrophysiological or behavioral, resulted from a post LPS-challenge-induced oxidative stress in the fetal hippocampus (as determined by tocopherol oxidation, protein carbonylation and GSH oxidation) that could be prevented by treatment with the antioxidant N-acetylcysteine given before [12] or after [11] the LPS challenge. Interestingly, oxidative stress and the electrophysiological and behavioral deficits only occurred in male animals [12]. Similar sex-specific effects are now frequently reported to result from exposure to immunogens during pregnancy [13], [14]. Prenatal LPS was also associated with a reduced expression of GluN1 NMDA receptor (NMDAr) subunit and a rapid decline in NMDAr-associated synaptic signals, together with a premature loss of long-term depression (LTD) and the transient occurrence of an aberrant form of LTP [15] in young male offspring.

Altered GABAergic inhibitory neurotransmission concomitant to reduced NMDAr-mediated neurotransmission led to altered excitatory/inhibitory (E/I) balance during postnatal development [16] and may contribute to defects in synaptic plasticity and the pathogenesis of neuropsychiatric diseases [17]. As recently reported, prenatal LPS challenge elicited a substantial deficit in hippocampal GABAergic neurons [7]. The functional consequence of such a deficit has not been evaluated. We reported in the present study that late gestational LPS challenge led to an impairment of the GABAergic transmission that is central to the dysregulation of synaptic plasticity observed in young offspring. Reinforcing GABAergic tone with tiagabine mostly prevented LTD deficit and the occurrence of abnormal LTP.

## Materials and Methods

### Study design

All experiments were carried out in accordance with the European Community Council Directive of November 24, 1986 (86/609/ECC). This study was approved by the local branch of the ‘Comité National de Réflexion Ethique sur l'Expérimentation Animale’ (CNREEA n°36) under the reference CEEA-LR-12099. All efforts were made to minimize animal suffering and to reduce the number of rats used.

Pregnant Sprague-Dawley rats (Centre d'Elevage Depré, St Doulchard, France) were used throughout this study. Animals were housed individually and randomly assigned to either of these experimental groups: (i) a group of control animals born to saline-injected dams (2 ml.kg^-1^ *ip* at the 19^th^ day of gestation GD19) (SAL animals) or (ii) a group of animals born to LPS-treated dams (500 µg.kg^−1^ *ip* at GD19) (LPS animals). LPS (from Escherichia coli, serotype O55∶B5) was obtained from Sigma-Aldrich (Saint-Quentin Fallavier, France). After birth, the size of the litters was restricted to ten pups. All animals were maintained on a 12-hr light/dark cycle with food and water *ad libitum*.

All procedures were performed on male offspring between 12 and 25 days old, before weaning and involved age-matched groups of animals. To avoid possible litter effects, groups of at least three independent litters were used for each parameter determination (stereology, electrophysiology, qRT-PCR).

A person blinded to allocation treatment analyzed the stereological data and electrophysiological recordings.

### Immunohistochemistry

The density of hippocampal GABAergic interneurons was studied between PD12 and PD18. Rats were deeply anesthetized (sodium pentobarbital, *ip*) and transcardially perfused with 4% paraformaldehyde. Brains were dissected and post-fixed overnight in 4% paraformaldehyde at 4°C. Sagittal brain sections (30 µm thick) were serially cut using a vibratome and stored at −20°C in 24-well plates containing a cryoprotective solution (30% glycerol, 30% ethylene glycol in 0.05 M PBS) until processed for immunostaining. Immunolabeling with the primary antibody mouse-anti glutamic acid decarboxylase (GAD)_67_ (Chemicon, Merck Millipore, Molsheim, France) was visualized using the streptavidin-biotin- peroxidase method as previously described [8]. The immunoreactive GAD_67_^+^ cell number was bilaterally determined on six to eight serial sections per animal, with an average intersection of 120 µm, containing the dorsal hippocampus [bregma 2.3 to 3.8 according to [18]]. Three regions in the hippocampal formation were defined: CA1, CA3 and the dentate gyrus (DG). The number of GAD_67_^+^ neurons was determined by unbiased stereological estimations using the Stereo Investigator (MicroBrightField, Williston, VT, USA) method combining an optical fractionation and a sampling fractionation. The immunoreactive cell density (total number of labeled cells counted/total area analyzed) was determined for each subject within a given subregion.

### Electrophysiology

After decapitation, brains were quickly dissected and placed in ice-cold buffer (124 mM NaCl, 3.5 mM KCl, 25 mM NaHCO_3_, 1.25 mM NaH_2_PO_4_, 1 mM CaCl_2_, 2 mM MgSO_4_, and 10 mM glucose bubbled with O_2_/CO_2_: 95/5%). Sagittal hippocampal slices (350 µm) were prepared with a vibratome (VT1000S Leica) and maintained at room temperature for at least 1 hr in the same buffer containing 2 mM CaCl_2_ also used for further recordings (extracellular buffer), as described [11], [12].

Slices were transferred to the recording chamber of an upright microscope (DMLFS Leica) and continuously superfused with the extracellular medium bubbled with O_2_/CO_2_ (95%/5%). Experiments were carried out at 32°C by warming the extracellular medium (Harvard Apparatus).

Inhibitory currents were measured by whole-cell patch-clamp recordings of CA1 hippocampal neurons, with glass microelectrodes (4–5 MΩ) filled with a solution comprising 130 mM CsMeSO_3_, 1 mM NaCl, 1 mM MgCl_2_, 1 mM EGTA, 5 mM N-(2,6 di-methyl-phenyl-carbamoyl-methyl)triethylammonium bromide (QX-314), 5 mM Hepes (pH 7.3), 4 mM Mg-adenosine 5′-triphosphate (Mg-ATP) and 0.3 mM Na-guanosine 5′-triphosphate (Na-GTP). In these conditions, the chloride ion reversal potential was approximately −70 mV. Thus, holding voltage at a 0 mV allowed to detect spontaneous or evoked GABA_A_ receptor-mediated postsynaptic currents (sIPSCs and eIPSCs, respectively) as outwards currents, which were recorded in the presence of D-(-)-2-amino-5-phosphonopentanoic acid (20 µM; D-AP5, Abcam) and 2,3-dihydroxy-6-nitro-7-sulfamoyl-benzo[f]quinoxaline-2,3-dione (10 µM; NBQX, Abcam). Intracellular Cs^+^ ions inhibited postsynaptic GABA_B_ receptor-mediated synaptic events. Access resistance was continually monitored and neuron records discarded if this parameter changed by more than 20%. Miniature IPSCs (mIPSCs) were recorded after a 30-min incubation with tetrodotoxin (100 nM; TTX, Tocris Bioscience).

To evoke IPSCs, a bipolar electrode connected to a stimulator (STG-1004, Multi Channel Systems) was positioned in the Schaffer collateral/commissural fibers at a distance of 0.5 mm from the recording electrode. Synaptic signals were obtained by delivering current pulses of fixed duration (100 µs) with varying intensity (from 3 to 400 µA). Decay kinetics of eIPSCs was determined by calculating the time constant tau using a single-exponential fitting. Input/output relationship was constructed by gradually increasing the stimulation intensity from a threshold stimulus determined in each individual experiment. Amplitudes of eIPSCs were further normalized to the capacitance of the recorded cell, calculated after analyzing capacitive currents elicited by the application of a −10 mV step to monitor access resistance. For each stimulation intensity, amplitudes of ten eIPSCs were averaged.

Extracellular recording of field excitatory synaptic transmission (fEPSP) were performed to measure long-term effects of low frequency stimulation (LFS, 1 stimulation/second for 15 min) and paired-pulse low frequency stimulation (ppulse-LFS, 1 paired-pulse stimulation/second for 15 min, with an inter-pulse interval of 50 ms), as previously described [12], [15]. A deficit in GABAergic inhibition may contribute to an enhanced propensity for epileptiform bursts during the early stages of postnatal development [19]. However, in our hands, slices from LPS rats, were not more susceptible than those from SAL rats to become epileptic.

The effects of tiagabine on LFS and ppulse-LFS-induced synaptic plasticity were compared between SAL and LPS groups. Tiagabine (20 µM, Abcam Biochemicals) was delivered in the perfusate during the recording of baseline activity and of the LFS or ppulse-LFS protocols. Preliminary experiments carried out by patch-clamp showed that this dose of tiagabine allowed a long-lasting blockade of GABA reuptake, persisting despite rinsing the extracellular buffer for an hour.

Postsynaptic currents and fEPSPs were measured with a patch-clamp amplifier, digitized and analyzed as described [11], [12].

qRT-PCR procedure and Paired-pulse depression (PPD) analysis are described in *Methods S1*.

### Statistics

Prism software (GraphPad Prism 5.0; GraphPad Software San Diego CA) was used to compare both the non linear regressions of the input/output curves, and the decay kinetics of post-synaptic inhibitory currents for both groups of animals. SigmaPlot 12 software (SigmaPlot 12.2; Systat Software San Jose CA) was used to analyze all other data. Quantitative variables following a Gaussian distribution were expressed as means ± SEM and intergroup comparisons were made with a two-tailed t-test. Quantitative variables that did not follow a Gaussian distribution were expressed as median and quartiles and intergroup comparisons were made with a Mann-Whitney rank sum test. After a preliminary F test for equality of variance, two-way repeated measures ANOVA followed by a Bonferroni post-hoc test was used to compare the GAD_67_^+^ cell densities in the different areas of the hippocampus. A Holm-Sidak post-hoc analysis was used to compare long-term plasticity between the different experimental groups. Probabilities of p<0.05 were considered significant.

## Results

### Prenatal LPS induced inflammation decreased the density of hippocampal GABAergic interneurons

The estimated volumes and the stereological parameters were not significantly different between SAL and LPS groups (*Table S1*).

**Figure 1:**
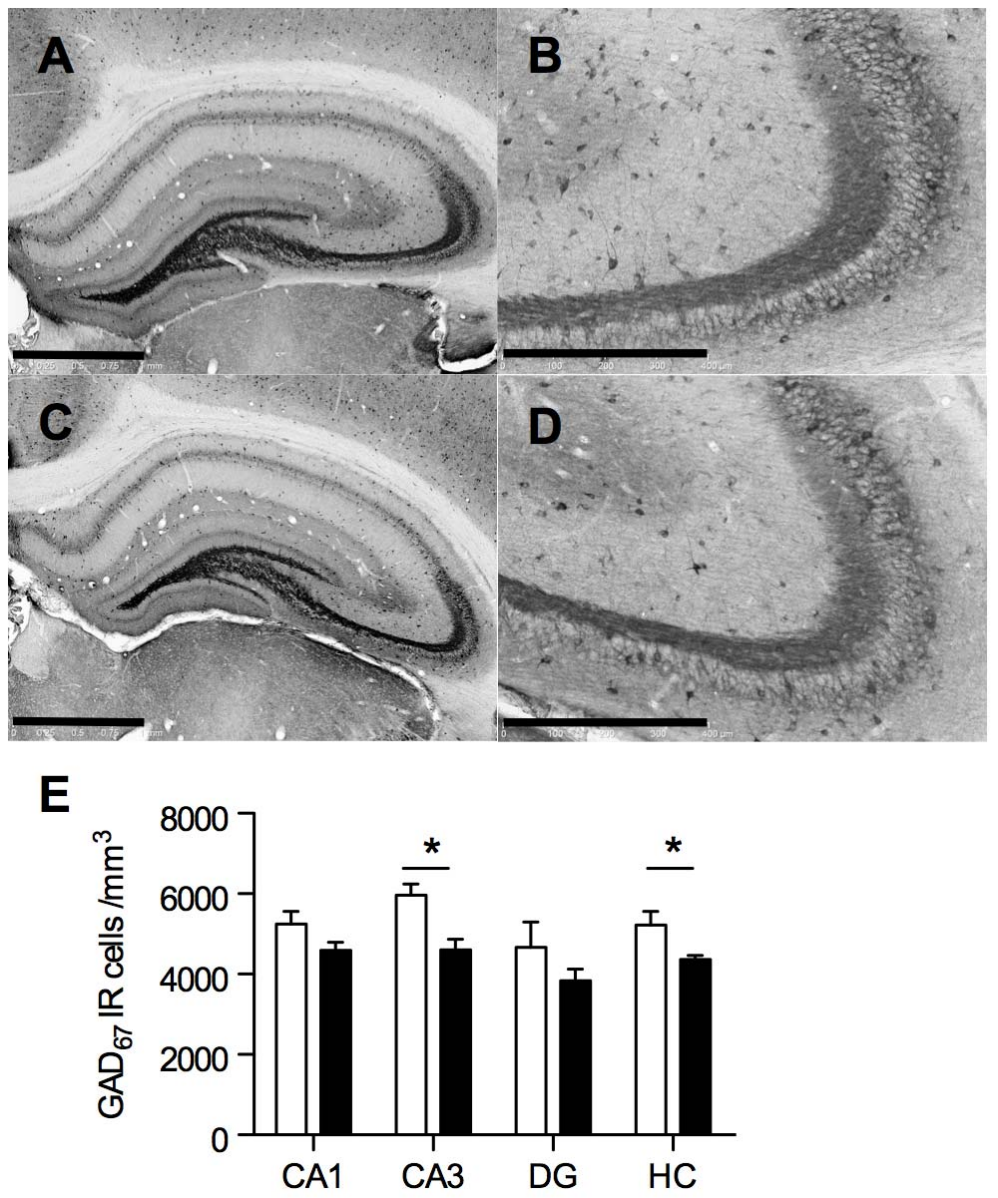
Prenatal LPS decreased GABAergic interneuron density in the hippocampus. Representative coronal sections of the whole hippocampus and of the CA3 area of 18-day-old SAL (**A,B**) and LPS animals (**C,D**) immunolabeled with anti-GAD_67_ antibody. Scale bar (A,C)  = 2 mm, Scale bar (B,D)  =  1 mm. (**E**) Late gestational LPS injection significantly decreased GAD_67_^+^ cell density in the hippocampus and notably in the CA3 area. Quantification of GAD_67_^+^ neurons has been performed as described in “Materials and Methods” from 4 animals per group. Data are presented as means ± SEM. ^*^p<0.05, for the comparison of SAL (open bars) *vs* LPS (black bars) using two-way ANOVA followed by Bonferroni's post-test. HC, hippocampus; DG, dentate gyrus.

There was a main effect of prenatal LPS treatment on hippocampal GAD_67_^+^ neuronal density of male offspring (F_1,24_ = 15; p<0.001), irrespective of the hippocampal area considered (F_3,24_ = 0.39; p = 0.760). Post hoc analyses showed that the deficit was significant in the CA3 area (Figure 1).

As we previously reported dramatic changes of synaptic properties in the CA1 region after prenatal LPS challenge, we next focused on this area to evaluate whether these changes involved a disturbance of GABAergic transmission [11], [12], [15].

### Prenatal LPS impaired GABAergic transmission in CA1

#### Impaired evoked GABAergic transmission

**Figure 2:**
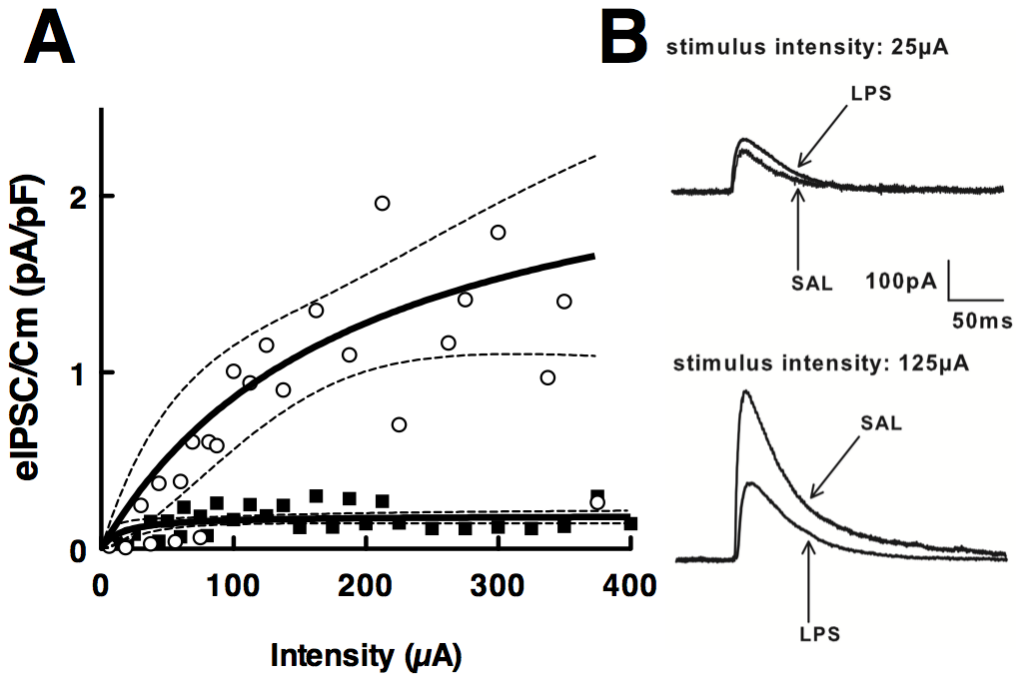
Prenatal LPS induced deficits in evoked inhibitory synaptic transmission in CA1 neurons. (**A**) I/O relationships of eIPSCs were established for both SAL and LPS rats. The amplitudes of eIPSCs were normalized to the capacitance of the recorded cell and plotted against corresponding stimulus intensities. The duration of the stimuli was fixed at 100 µs. Hyperbolic fitting was performed on 127 experimental points obtained from SAL (open circles; N = 9) animals and 120 experimental points obtained from LPS (filled squares; N = 9) animals. Stippled zones represent the 95% Confidence Intervals of the curves. Each point represents the mean amplitude for the corresponding intensity of stimulation. For readability, SEMs were not represented. * p<0.05, for the comparison of LPS vs SAL animals, using two-way ANOVA followed by Holm-Sidak post-hoc analysis. (**B**) Representative traces of the eIPSCs recorded in SAL and LPS animals at two different intensities.

Evoked inhibitory postsynaptic currents (eIPSCs) were measured in the CA1 area of SAL and LPS rats. In SAL animals, eIPSC amplitudes increased as a function of stimulus intensity, best fitted with a hyperbolic relationship (N = 9 rats; 127 data points), even when no plateau could be reached at the maximum intensity tested (Figure 2). In LPS rats, eIPSC amplitudes followed a similar hyperbolic relationship (N = 9 rats; 120 data points), but the saturating plateau was reached as soon as a small intensity was applied, and its amplitude was significantly dampened compared with those in SAL animals. Non linear regression analysis of the data evidenced that best fitted curves were significantly different for SAL and LPS animals (F_2,243_ = 29.31; p<0.001).

#### Impaired spontaneous GABAergic transmission

**Figure 3:**
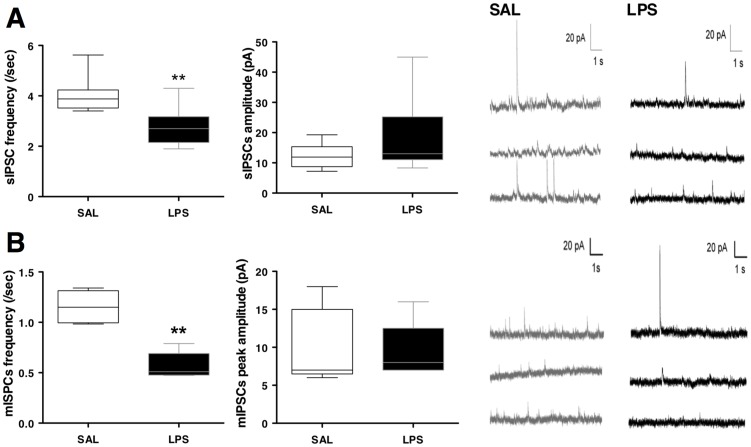
Prenatal LPS-induced deficits in spontaneous and miniature GABAergic activities in CA1 pyramidal cells. (**A**) Spontaneous GABAergic activity recorded in CA1 pyramidal cells. The recapitulative graphs plot mean sIPSC frequency (left) and amplitude (right). The boxes define the median, 25^th^ and 75^th^ percentiles. The whiskers represent 10^th^ and 90^th^ percentiles. ^**^ p<0.01, Mann and Whitney rank sum test, N = 8 animals per group. (**B**) Miniature GABAergic activity recorded in CA1 pyramidal cells in the presence of TTX (100 nM). The recapitulative graphs plot mean mIPSC frequency (left) and amplitude (right). The boxes define the median, 25^th^ and 75^th^ percentiles. The whiskers represent the 10^th^ and 90^th^ percentiles. ^**^ p<0.01, Mann and Whitney rank sum test, N = 5 animals per group. Representative traces of GABAergic spontaneous activity (top) and GABAergic miniature activity (bottom) are shown in individual neurons from SAL (gray) and LPS (black) rats.

Spontaneous inhibitory postsynaptic currents (sIPSCs) were measured in the CA1 of SAL and LPS rats (N = 8 per group). The frequency of sIPSCs was significantly decreased in LPS compared with SAL animals. By contrast, sIPSCs mean amplitude did not differ between groups (Figure 3A). This suggested that the prenatal LPS challenge impaired spontaneous GABA release from synaptic terminals, without postsynaptic changes.

#### Impaired miniature GABAergic transmission

To confirm this, miniature inhibitory postsynaptic currents (mIPSCs) were recorded in the presence of TTX to block the network-dependent activity. Whereas the mean frequency of mIPSC was significantly reduced in LPS rats, their mean amplitude did not differ from that observed in SAL animals (Figure 3B). This lower frequency of mIPSCs confirmed a presynaptic deficit of GABAergic transmission in LPS animals.

The similar amplitude of mIPSCs suggested that vesicular refilling of GABA was not affected by LPS prenatal inflammation. Consistently, the expressions of the major membrane GABA transporter (GAT), GAT1, mRNA [20] and most particularly of the GABA vesicular transporter (VGAT) mRNA were not significantly different between SAL and LPS animals (*Figure S1*). The activity of GABA transporter was further appreciated by evaluating the decay kinetics of eIPSCs. Single-exponential fitting of the time course of the eIPSCs yielded the time constant tau, which was similar in SAL and LPS animals (98.2±11.0 ms; N = 22 SAL vs 119.2±13.0 ms; N = 24 LPS; p = 0.110), independently of the stimulus intensity applied. Changes in tau values were next estimated to evaluate the effect of tiagabine, a selective GAT-1 inhibitor, in both SAL and LPS rats. Preliminary experiments had shown that 20 µM tiagabine induced a long-lasting blockade of GABA reuptake, persisting one-hour after washout. Tiagabine slowed down the decay kinetics of eIPSCs in both groups, thus enhancing the tau value. Ratios between tau values after and before tiagabine perifusion were comparable in SAL and LPS groups: 230±20 % (N = 12) and 223±16 % (N = 7), respectively (p = 0.805; *Figure S1*). Similar results were obtained using another less selective GAT1 inhibitor, nipecotic acid (not shown), albeit at higher concentrations (100–300 µM). This suggested a similar GAT-1 efficiency in SAL and LPS rats.

The decay constant of mIPSCs was not significantly different between SAL and LPS animals (40.8±11.6 ms *vs* 20.7±4.2 ms; p = 0.222; N = 5 per group). The rise time constant of mIPSCs was not significantly different between SAL and LPS animals (17.0±5.6 ms *vs* 8.3±0.7 ms; p = 0.222; N = 5 per group). Thus, it was unlikely that LPS prenatal inflammation would modify postsynaptic GABA_A_ receptor properties in young animals. Consistently, expressions of the α2, α3 and α5 subunits of the GABA_A_ receptor were similar in SAL and LPS animals (*Figure S2*). This agrees with a predominant presynaptic impairment after prenatal LPS challenge, leading to the decrease of GABA spontaneously released from synaptic terminals, without postsynaptic changes.

Release of GABA may be controlled by presynaptic metabotropic presynaptic GABA_B_ receptors [21]. To test the activity of these receptors, we studied the GABA_B_ receptor-dependent mechanism of paired pulse depression (PPD) of IPSCs [22]. Similar PPD of sIPSCs were observed in both SAL and LPS animals (*Figure S3*). As expected, PPD decreased as far as the inter-pulse interval increased and the application of the GABA_B_ receptor antagonist CGP 55845 limited this PPD. This effect was not significantly different between SAL and LPS rats, suggesting that prenatal LPS did not affect GABA_B_ receptor-mediated control of GABA release.

As GABA is a key modulator of LTD of glutamatergic transmission after stimulation of Schaffer collaterals [23], we next hypothesized that compensating the deficit of GABA release by reinforcing GABAergic tone with the GAT1 antagonist tiagabine may prevent the LTD deficit in young LPS animals.

### GAT1 inhibition by tiagabine prevented LTD impairment after low frequency stimulation (LFS) in LPS animals

**Figure 4:**
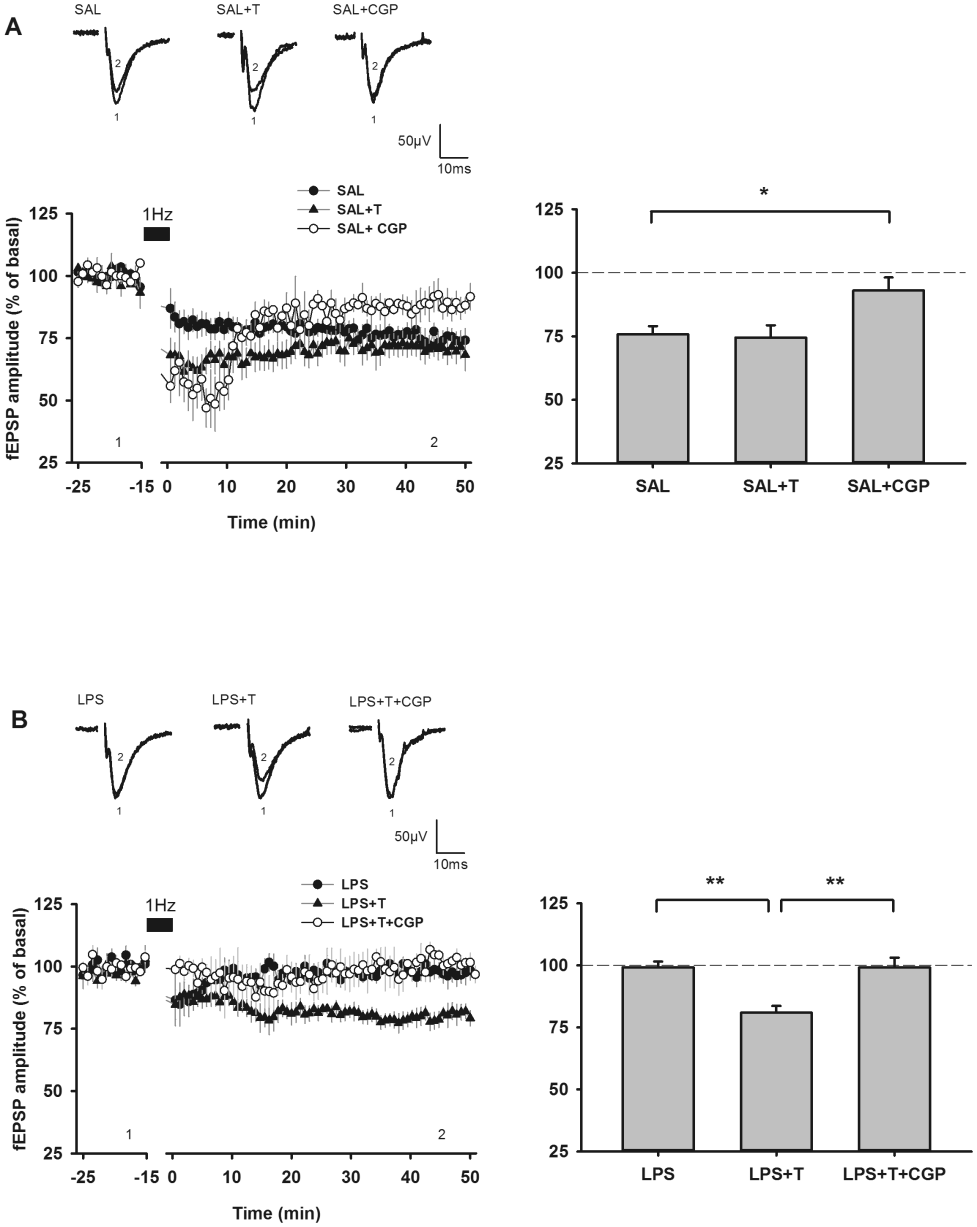
Tiagabine restored LTD via the activation of GABA_B_ receptors in LPS animals. Tiagabine (20 µM) and/or CGP55845 (1 µM) were applied in the perfusate during both the recording of baseline activity and LFS (1 Hz stimulation, 15 min) delivery. (**A**) Time-course and recapitulative graph depicting LTD induction in control (SAL) animals. LFS induced an LTD of fEPSP amplitude in control animals (SAL; filled circles; N = 8), which was significantly blocked by the GABA_B_ receptor antagonist CGP55845 (SAL+CGP; open circles; N = 5; * p<0.05 vs SAL group). Tiagabine had no significant effect on LTD level (SAL+T; filled triangles; N = 8). (**B**) Time-course and recapitulative graph depicting LTD induction in LPS animals. LFS did not trigger LTD in LPS animals (LPS; filled circles, N = 8), while tiagabine restored the LTD occurrence (LPS+T; filled triangles; N = 7; ** p<0.01 vs LPS group). The effect of tiagabine was not observed when co-applied with CGP55845 (LPS+T+CGP; open circles; N = 5; ** p<0.01 vs LPS+T group). Time-courses are illustrated by representative traces of fEPSP recorded in each group and extracted both before (1) and 45 min following (2) the stimulation train as indicated by the time-points on the graphs. On the right, histograms recapitulate mean fEPSP amplitudes (± SEM) recorded under different conditions. Data are fEPSP amplitudes averaged between time 20 min and time 50 min for each animal (see A or B graph legends for respective N). The statistical significance of the differences between groups was assessed by performing pairwise multiple comparison Holm Sidak analysis.

The long-term effects of LFS differed, depending on both the prenatal challenge and the presence or the absence of tiagabine (F_5,41_ = 8.13; p<0.0001) (Figure 4A). In SAL rats, LFS of Schaffer collaterals triggered a long-term depression of field excitatory postsynaptic potential (fEPSP) amplitude (LFS-LTD): 76.7±3.0 % of basal fEPSP amplitude (N = 8), that was significantly (p<0.05), but only transiently enhanced in tiagabine perfused slices. On the long-term, tiagabine did not modulate LFS-LTD in these animals (71.4±4.3 % of basal fEPSP amplitude; N = 8). As expected, LFS-LTD in these animals was dependent on the activation of GABA_B_ receptors [23]. The application of CGP 55845 (1 µM) also promoted a pronounced but transient depression, but prevented the occurrence of LFS-LTP (89.5±5.1 % of basal fEPSP amplitude; N = 5) (Figure 4A). As previously shown [15], no LFS-LTD was observed in LPS rats (98.6±2.3 % of basal fEPSP amplitude; N = 8). The effect of prenatal LPS on LTD induction was significant (p<0.001). Tiagabine allowed LFS to trigger LTD in LPS rats (81.3±2.6 % of basal fEPSP amplitude; N = 7) (Figure 4B). The restoring effect of tiagabine was also dependent on GABA_B_ receptors, as it was fully abolished in the presence of CGP 55845 (99.1±4.1 % of basal fEPSP amplitude; N = 5) (Figure 4B).

### GAT1 inhibition by tiagabine prevented abnormal LTP after paired-pulse low frequency stimulation (ppulse-LFS) in LPS animals

**Figure 5:**
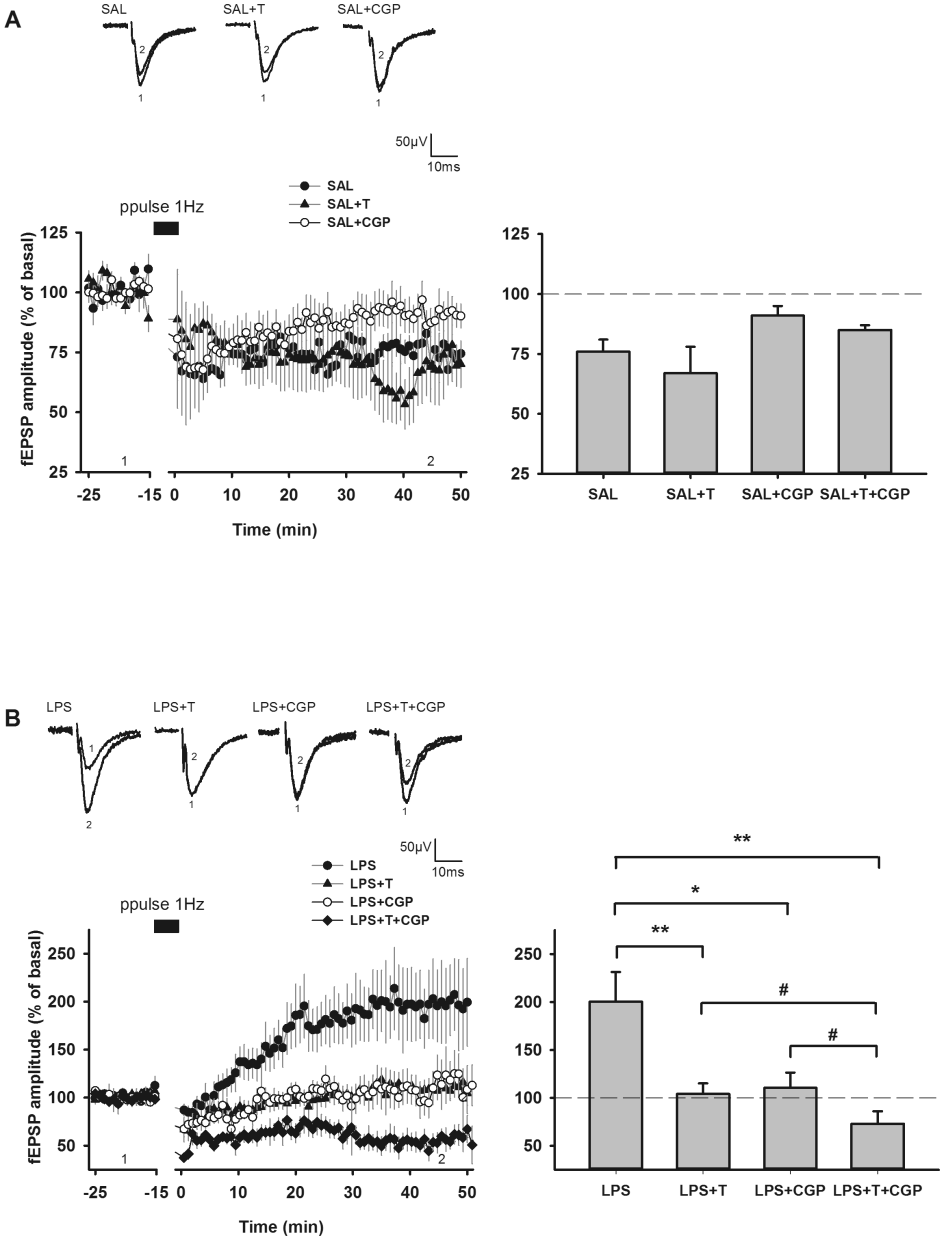
Tiagabine normalized aberrant synaptic plasticity phenomena in young LPS animals. Tiagabine (20 µM) and/or CGP55845 (1 µM) were applied in the perfusate during both the recording of baseline activity and ppulse-LFS (paired-pulse 1 Hz stimulation, 50 ms inter-pulse interval, 15 min) delivery. (**A**) Time-course and recapitulative graph depicting LTD induction in control (SAL) animals. Ppulse-LFS induced an LTD of fEPSP amplitude in control animals (SAL; filled circles; N = 7), which was partially blocked by the GABA_B_ receptor antagonist CGP55845 (SAL+CGP; open circles; N = 9). Tiagabine had no significant effect on LTD level either in the absence (SAL+T; filled triangles; N = 8) or in the presence of CGP55845 (N = 6). (**B**) Time-course and recapitulative graph depicting LTD induction in LPS animals. Ppulse LFS application led to the occurrence of a slow-onset LTP in LPS offspring (LPS; filled circles; N = 13), which was not observed in the presence of either tiagabine (LPS+T; filled triangles; N = 11; ** p<0.01 vs LPS group) or CGP55845 (LPS+C; open circles; N = 6; * p<0.05 vs LPS group). LTD could be obtained after ppulse-LFS in the concurrent presence of tiagabine and CGP (LPS+T+CGP; filled diamonds; N = 6; ** p<0.01 vs LPS group). Under these latter conditions, the average of fEPSP amplitudes was significantly different the one obtained in the presence of either tiagabine (# p<0.05 vs LPS+T group) or CGP (# p<0.05 vs LPS+C group) after ppulse-LFS application. Time-courses are illustrated by representative traces of fEPSP recorded in each group and extracted both before (1) and 45 min following (2) the stimulation train as indicated by the time-points on the graphs. On the right, histograms recapitulate mean fEPSP amplitudes (± SEM) recorded under different conditions. Data are fEPSP amplitudes averaged between time 20 min and time 50 min for each animal (see A or B graph legends for respective N). The statistical significance of the differences between groups was assessed by performing pairwise multiple comparison Holm Sidak analysis.

The long-term effects of ppulse-LFS also differed, depending on the prenatal challenge, the presence of tiagabine or of CGP 55845 (F_7,65_ = 6.71; p<0.0001). In SAL rats, ppulse-LFS of Schaffer collaterals triggered a long-term depression of the fEPSP amplitude (ppulse-LTD): 75.1±4.4 % of basal fEPSP amplitude (N = 7), that was not modified in the presence of tiagabine (67.1±10.7 % of basal fEPSP amplitude; N = 8), but that was inhibited, although non significantly, in the presence of CGP 55845 (91.1±3.8 % of basal fEPSP amplitude; N = 9; p = 0.07). Perfusion with both tiagabine and CGP 55485 resulted in a similar LTD as in control SAL animals (84.9±2.1 % of basal fEPSP amplitude; N = 6; p = 0.07) (Figure 5A). In LPS rats, as previously shown [15], ppulse-LFS of Schaffer collaterals induced a long-term potentiation of the fEPSP amplitude (ppulse-LTP) (195.4±28.0 % of basal fEPSP amplitude; N = 13). The effect of prenatal LPS on ppulse-induced glutamatergic synaptic plasticity was significant (p = 0.002). Perfusion with tiagabine (103.7±10.0 % of basal fEPSP amplitude; N = 11), or perfusion with CGP 55845 (110.4±16.2 % of basal fEPSP amplitude; N = 6) prevented the aberrant ppulse-LTP in LPS rats. Whereas none of these agents were able to restore a normal response in these animals when used separately, their co-application actually restored a full ppulse-LTD (72.6±13.7 % of basal fEPSP amplitude; N = 6; p<0.05) (Figure 5B).

## Discussion

Late gestational LPS induced inflammation led to a deficit in GABAergic neurons in the hippocampus, which was associated with impaired GABA release and decreased inhibitory transmission in the CA1 area. Pharmacological enhancement of GABAergic tone with tiagabine prevented the LFS-LTD deficit and prevented the occurrence of the abnormal ppulse-LTP. We thus confirmed that the decrease in GABAergic input onto pyramidal neurons contributed to the abnormal synaptic plasticity observed in LPS young offspring.

Although the density of hippocampal GABAergic interneurons was slightly reduced in each area of the hippocampus, the decrease was only significant in CA3. This seemed to be relevant with regard to postmortem studies performed on schizophrenic and bipolar patients, which demonstrated a predominant remodeling of the GABAergic system in the CA2/CA3 area [24]–[27].

Nevertheless the present study provided evidence that GABA transmission was altered in CA1 and had functional consequences on synaptic plasticity in CA1 pyramidal neurons. We observed considerable lessening of sIPSC and mIPSC frequency in LPS rats, consistent with a presynaptic impairment of GABAergic transmission, most probably unrelated to a local deficit of GABA synthesis, as the expression of GAD_65_, which is important for local control of GABA synthesis at the synaptic sites remained unchanged in LPS animals (*Figure S4*). A reduced number of synaptic terminals associated with the decreased number of GABAergic interneurons in LPS animals may be involved in such impairment of GABA release.

The similar amplitude and kinetics of mIPSCs suggested that LPS prenatal inflammation did not affect vesicular refilling of GABA and did not induce any change in postsynaptic GABA_A_ receptor responsiveness in young offspring. This was further supported by the similar expression of VGAT, GAT-1, α2, α3 and α5 subunits of the GABA_A_ receptor between SAL and LPS animals, even though our experiments did not allow excluding further translational or post-translational modulation of these receptors. Conversely, an increase in GABA_A_ receptor α2 and α5 subunits expression was observed in adult animals prenatally exposed to the viral mimics polyinosinic∶polycytidylic acid (poly∶IC) and interleukin-6 respectively [28], [29]. This may indicate a compensatory response to decreased GABA release [30], which might appear late in adulthood in LPS animals.

To explore whether the GABA deficit contributed to the impaired LTD of glutamatergic transmission in young LPS offspring [15], we next evaluated the effect of GABAergic transmission enhancement on LFS and ppulse-LTD induction. Improved GABA transmission could be achieved either by enhancing synaptically released GABA through the blockade of GABA uptake or by acting on postsynaptic GABA_A_ receptors. Direct targeting of GABA_A_ receptors demonstrated limited efficiency in treating the cognitive impairments in schizophrenic patients [31] or unexpected toxicity [32]. Moreover, since GABA_A_ receptors are pentameric complexes of subunits, with specific brain regional expression that can be modified by pharmacological agents or under pathological conditions [33], appropriate targeting of GABA_A_ receptors may be challenging. We therefore chose to implement GABAergic tone with the GABA transporter inhibitor tiagabine. Tiagabine has already been approved as an anticonvulsant with slight side effects and is thus an interesting drug in a translational approach. In the present study, tiagabine similarly slowed down eIPSC decays in both SAL and LPS animals, which indicated that this molecule actually increased GABAergic tone in both animals.

We next evaluated the effect of tiagabine on LFS- and ppulse-LTD induction. LFS-LTD is mainly dependent on NMDAr activation [15], [34], [35], and GABA_B_ receptor activation may be required to induce NMDAr-dependent LTD in young animals [23]. Accordingly, enhancing the GABA tonus via tiagabine in the present experiments did not modify the LFS-LTD in SAL rat, while inactivation of GABA_B_ receptors resulted in a complete disappearance of LFS-LTD. LPS-induced prenatal inflammation results in an NMDAr dysfunction [11], [12], [15], [36] and accelerates the developmental decay of LFS-LTD [15]. While confirming these results, we demonstrated here that a simple tiagabine perfusion of hippocampal slices was sufficient to prevent the loss of LFS-LTD despite the reduced functionality of NMDAr, and that this restoring effect of tiagabine occurred via the activation of GABA_B_ receptors.

GAT1-mediated GABA transport regulates the electrophysiological effects of GABA_B_ receptor activation by synaptically-released GABA [20]. Thus, in LPS rats, the decreased synaptic release of GABA may result in the observed impairment of LFS-LTD. Blockade of GAT1 activity by tiagabine during repeated LFS stimulation may promote a small extracellular GABA build-up, sufficient to activate the GABA_B_ receptors located on presynaptic GABAergic terminals. The resulting disinhibition would have a strongly permissive role in NMDAr activation and facilitate the induction of NMDAr-mediated plasticity, as evidenced for LFS-LTD induction. In line with this hypothesis, a recent report indicated that baclofen, a GABA_B_ receptor agonist, improved neuronal excitability, gamma oscillations and behavior in NMDA-NR1^neo−/−^ mice that are completely devoid of the obligatory GluN1 subunit of the NMDAr from birth [37].

As LTD induced by distinct protocols vary in their mechanisms [38], slightly different mechanisms may be responsible for inhibiting the aberrant ppulse-LTP in LPS rats. Ppulse-LFS is supposed to mobilize group 1 glutamate metabotropic receptor (mGlu1/5r) to induce an LTD in normal animals [39]. We previously reported that the aberrant ppulse-LTP in PD16-25 LPS rats was insensitive to NMDA blockade, but completely inhibited by the application of MPEP, an mGlu1/5r antagonist. Nevertheless MPEP did not fully reinstate LTD normally obtained in SAL rats in this age range after ppulse delivery [15]. In a similar way, we observe here that tiagabine, as MPEP, prevented the aberrant ppulse-LTP without reverting it to a full ppulse-LTD.

Under a LFS protocol, the effect of tiagabine in LPS rats involves the activation of GABA_B_ receptors. By contrast, under a ppulse-LFS stimulation protocol, the effect of tiagabine is not simply dependent of the enhancement of GABA_B_ receptors activity. Unexpectedly indeed, inactivating the GABA_B_ receptors activity by itself prevented the aberrant ppulse-LTP, as did tiagabine application, without reverting it to a full ppulse-LTD. Only a concurrent perfusion of tiagabine and CGP 55845 was able to restore a normal synaptic phenotype in LPS rats. A possible explanation of this result would be that, under enhanced extracellular GABA concentrations, blockage of GABA_B_ receptors uncovered GABA_A_ receptor activation, thus facilitating LTD occurrence.

Taken together our data suggest that two possibly independent mechanisms implying GABA may be activated in LPS animals to promote the aberrant ppulse-LTP: (i) a reduced release of GABA, resulting in an enhanced stimulation of pyramidal neurons and (ii) an enhanced activation of postsynaptic GABA_B_ receptor located on other neurons (possibly on pyramidal neurons themselves). Further investigations are required to elucidate how these mechanisms could combine to convert LTD into LTP in LPS animals.

Summing up, LPS immune challenge in late gestation leads to early GABAergic dysregulation. The GABA reuptake inhibitor tiagabine restores a normal or sub-normal synaptic plasticity in young LPS male offspring, demonstrating that this inhibitory input decrease is involved in the impairment of glutamatergic synaptic plasticity. It remains to be determined whether acute or chronic tiagabine administration *in vivo* would also prevent the spatial working memory deficiency observed in LPS animals. Should this be the case, tiagabine, which has already been approved as an anticonvulsant with slight side effects, will be an interesting drug to prevent the cognitive defects associated with prenatal inflammation.

## Supporting Information

Figure S1 **GABA transporter expression and activity were unaffected by prenatal LPS.** (**A**) VGAT and GAT-1 mRNA expression was evaluated in SAL and LPS animals (N = 5 each). The boxes define the median, 25th and 75th percentiles. The whiskers represent 10th and 90th percentiles. (**B**) Recapitulative graph plotting averaged tau values obtained in the presence of tiagabine and normalized to baseline values in SAL (N = 12) and LPS (N = 7) rats. Tiagabine (20 µM) was applied in the perfusate while eIPSCs were recorded by holding membrane voltage at 0 mV, in the presence of ionotropic glutamate receptor antagonists. Tau values were obtained from single exponential fitting of the decay time-course of eIPSCs. (**C**) Illustrative traces depict the slowing-down of eIPSC decay elicited by tiagabine in SAL (top traces) and LPS (bottom traces) rats.
(PDF)

Figure S2 **α2, α3, α5 GABAA-receptor subunits' expression was unaffected by prenatal LPS.** Means and SEM are represented. N = 5 animals for SAL (open bar) and LPS (black bar) groups.
(PDF)

Figure S3 **Paired-pulse depression of eIPSCs.** Two shocks were delivered with increasing inter-pulse intervals of 100, 200, 300 and 400 ms. (**A**) Representative eIPSC traces illustrating paired-pulse depression and its blockade by CGP 55845 in both SAL (in gray) and LPS (in black) rats are shown. The amplitude of the eIPSC evoked by the second stimulation (A2) was normalized to the amplitude of the first one (A1) and expressed as a percentage. (**B**) This percentage was below 100%, indicating a paired-pulse depression. N = 13 for SAL animals (open bars), N = 12 for LPS animals (black bars). (**C**) Antagonizing GABA_B_ receptors with 1 µM CGP 55845 limited paired-pulse depression in both SAL and LPS animals, resulting in an increase in the A2/A1 ratio. Percentages of increase in A2/A1 ratios induced by the perifusion of 1 µM CGP 55845 were not significantly different between SAL and LPS animals. N = 4 animals per group. Data shown are means ± SEM.
(PDF)

Figure S4 **Expression of GAD65 and GAD67 in SAL or LPS-treated rats.** GAD67 mRNA expression was normalized to β actin and GAPDH mRNA expression (reference genes). N = 5 animals for SAL (open bar) and LPS (black bar) groups.
(PDF)

Table S1 **Stereological parameters.** Values are means ± SEM (N = 4 per group). A Mann and Whitney rank sum test was used.
(PDF)

Methods S1 Specific methods used for obtaining supporting information. qRT-PCR. Paired-pulse depression analysis.
(DOCX)
