# Supplementary material for: Tiagabine Improves Hippocampal Long-Term Depression in Rat Pups Subjected to Prenatal Inflammation
Source: PLoS One. 2014 Sep 3;9(9):e106302. doi: 10.1371/journal.pone.0106302 (PMC4153642; doi:10.1371/journal.pone.0106302)
Supplement: Figure S1 — GABA transporter expression and activity were unaffected by prenatal LPS. (A) VGAT and GAT-1 mRNA expression was evaluated in SAL and LPS animals (N = 5 each). The boxes define the median, 25th and 75th percentiles. The whiskers represent 10th and 90th percentiles. (B) Recapitulative graph plotting averaged tau values obtained in the presence of tiagabine and normalized to baseline values in SAL (N = 12) and LPS (N = 7) rats. Tiagabine (20 µM) was applied in the perfusate while eIPSCs were recorded by holding membrane voltage at 0 mV, in the presence of ionotropic glutamate receptor antagonists. Tau values were obtained from single exponential fitting of the decay time-course of eIPSCs. (C) Illustrative traces depict the slowing-down of eIPSC decay elicited by tiagabine in SAL (top traces) and LPS (bottom traces) rats. (PDF) [file pone.0106302.s001.pdf]

Supporting Figure S1: GABA transporter expression and activity were unaffected by prenatal LPS.

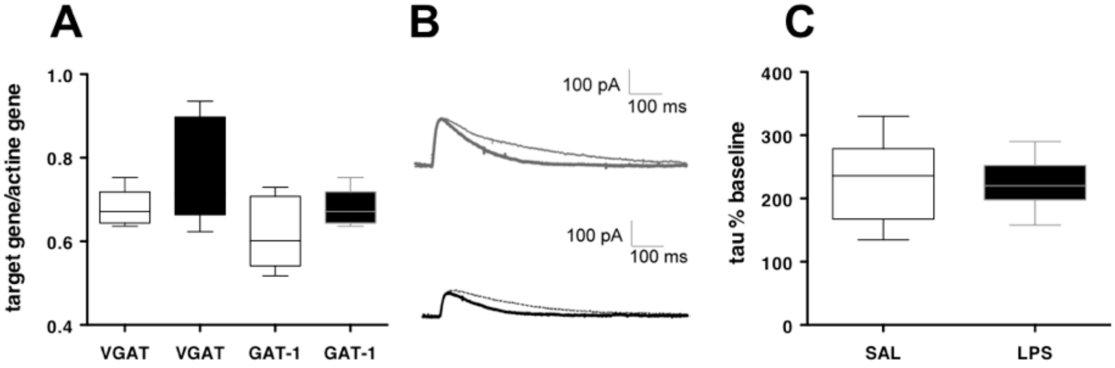

(**A**) VGAT and GAT-1 mRNA expressions were evaluated in SAL (open boxes) and LPS (black boxes) animals (N = 5 each) by qRT-PC. The boxes define the median, 25th and 75th percentiles. The whiskers represent 10th and 90th percentiles. (**B**) Illustrative traces depict the slowing-down of eIPSC decay elicited by tiagabine in SAL (top traces) and LPS (bottom traces) rats. Tiagabine (20 µM) was applied in the perfusate while eIPSCs were recorded by holding membrane voltage at 0 mV, in the presence of ionotropic glutamate receptor antagonists. Single exponential fitting of the decay time-course of eIPSCs allows determining tau values in the various experimental conditions. (**C**) Recapitulative graph plotting averaged tau values obtained in the presence of tiagabine and normalized to those obtained in the absence of tiagabine in both SAL (N = 12) and LPS (N = 7) rats.
