# Supplementary material for: Tiagabine Improves Hippocampal Long-Term Depression in Rat Pups Subjected to Prenatal Inflammation
Source: PLoS One. 2014 Sep 3;9(9):e106302. doi: 10.1371/journal.pone.0106302 (PMC4153642; doi:10.1371/journal.pone.0106302)
Supplement: Figure S2 — α2, α3, α5 GABAA-receptor subunits' expression was unaffected by prenatal LPS. Means and SEM are represented. N = 5 animals for SAL (open bar) and LPS (black bar) groups. (PDF) [file pone.0106302.s002.pdf]

Supporting Figure S2: α2, α3, α5 $GABA_A$-receptor subunits' expression was unaffected by prenatal LPS.

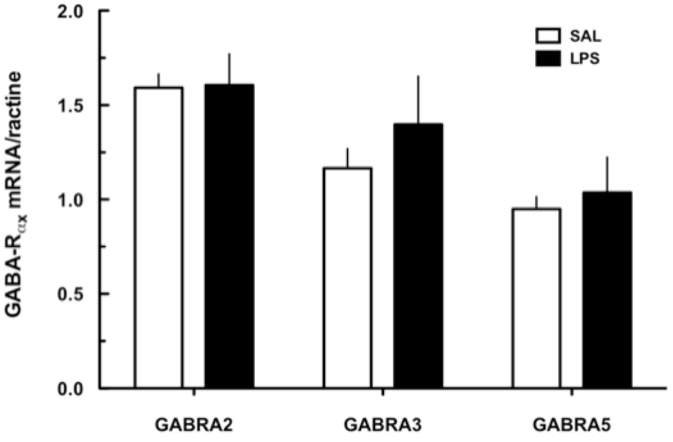

mRNA expressions of $GABA_A$-receptor subunits was determined by qRT-PCR in both SAL (open bar) and LPS (black bar) rats (N = 5 animals in each group). Means and SEM are represented.
