# Supplementary material for: Tiagabine Improves Hippocampal Long-Term Depression in Rat Pups Subjected to Prenatal Inflammation
Source: PLoS One. 2014 Sep 3;9(9):e106302. doi: 10.1371/journal.pone.0106302 (PMC4153642; doi:10.1371/journal.pone.0106302)
Supplement: Figure S3 — Paired-pulse depression of eIPSCs. Two shocks were delivered with increasing inter-pulse intervals of 100, 200, 300 and 400 ms. (A) Representative eIPSC traces illustrating paired-pulse depression and its blockade by CGP 55845 in both SAL (in gray) and LPS (in black) rats are shown. The amplitude of the eIPSC evoked by the second stimulation (A2) was normalized to the amplitude of the first one (A1) and expressed as a percentage. (B) This percentage was below 100%, indicating a paired-pulse depression. N = 13 for SAL animals (open bars), N = 12 for LPS animals (black bars). (C) Antagonizing GABAB receptors with 1 µM CGP 55845 limited paired-pulse depression in both SAL and LPS animals, resulting in an increase in the A2/A1 ratio. Percentages of increase in A2/A1 ratios induced by the perifusion of 1 µM CGP 55845 were not significantly different between SAL and LPS animals. N = 4 animals per group. Data shown are means ± SEM. (PDF) [file pone.0106302.s003.pdf]

Supporting Figure S3: Paired-pulse depression (PPD) of eIPSCs

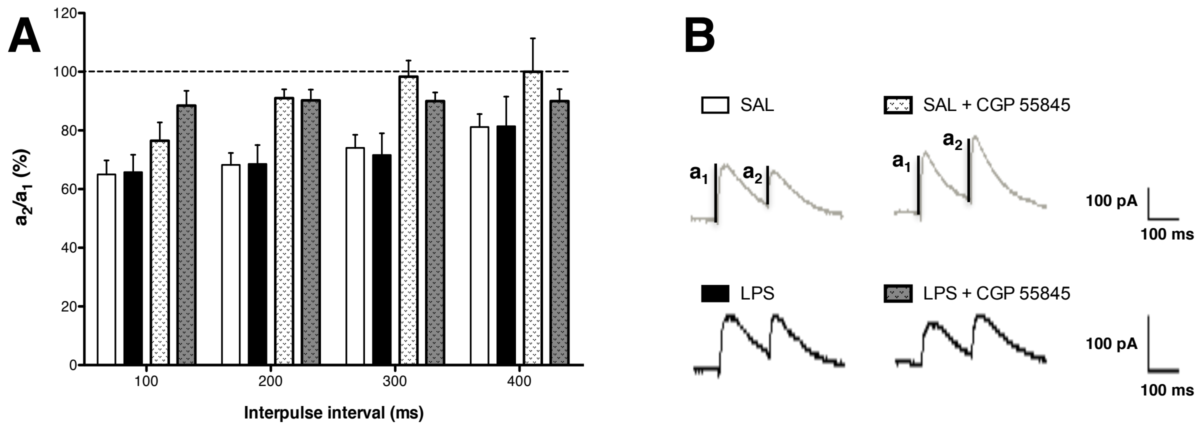

Two shocks were delivered with increasing inter-pulse intervals of 100, 200, 300 and 400 ms to induce PPD. (**A**) The amplitude of the eIPSC evoked by the second stimulation (a_2) was normalized to the amplitude of the first one (a_1) and expressed as a percentage. This percentage was below 100%, indicating a PPD, which decreased as the inter-pulse interval increased. N = 13 for SAL animals (open bars), N = 12 for LPS animals (black bars). (**B**) Representative eIPSC traces illustrating paired-pulse depression and its blockade by CGP 55845 in both SAL (in gray) and LPS (in black) rats are shown.

Antagonizing $GABA_B$ receptors with 1 μM CGP 55845 lessened paired-pulse depression in both SAL and LPS animals, resulting in an increase in the a_2/a_1 ratio. Percentages of increase in a_2/a_1 ratios induced by the perifusion of 1 μM CGP 55845 were not significantly different between SAL and LPS animals. N = 4 animals per group. Data shown are means ± SEM.
