# Supplementary material for: Tiagabine Improves Hippocampal Long-Term Depression in Rat Pups Subjected to Prenatal Inflammation
Source: PLoS One. 2014 Sep 3;9(9):e106302. doi: 10.1371/journal.pone.0106302 (PMC4153642; doi:10.1371/journal.pone.0106302)
Supplement: Figure S4 — Expression of GAD65 and GAD67 in SAL or LPS-treated rats. GAD67 mRNA expression was normalized to β actin and GAPDH mRNA expression (reference genes). N = 5 animals for SAL (open bar) and LPS (black bar) groups. (PDF) [file pone.0106302.s004.pdf]

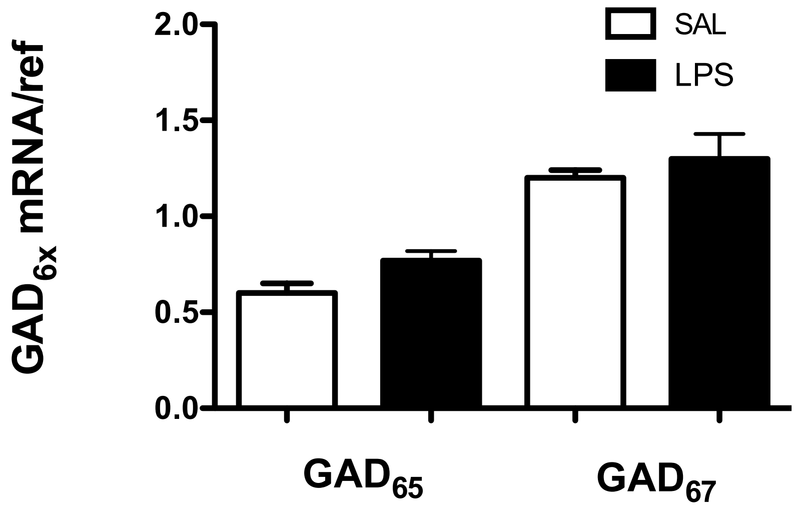

Figure S4. Expression of GAD_{65} and GAD_{67} in SAL or LPS-treated rats. GAD_{67} mRNA expression was normalized to β actin and GAPDH mRNA expression (reference genes).
N = 5 animals for SAL (open bar) and LPS (black bar) groups.
