# Supplementary material for: Tiagabine Improves Hippocampal Long-Term Depression in Rat Pups Subjected to Prenatal Inflammation
Source: PLoS One. 2014 Sep 3;9(9):e106302. doi: 10.1371/journal.pone.0106302 (PMC4153642; doi:10.1371/journal.pone.0106302)
Supplement: Table S1 — Stereological parameters. Values are means ± SEM (N = 4 per group). A Mann and Whitney rank sum test was used. (PDF) [file pone.0106302.s005.pdf]

Supporting Table 1

Area		Nb slices	Thickness (µm)	Estimated volume (mm^3)	Nb sampling zones	Nb neurons
HC	SAL	6.5 ± 0.3	14.3 ± 0.3	6.5 ± 0.4	257.5 ± 11.2	3986 ± 55
	LPS	7.0 ± 0.4	13.7 ± 0.5	7.0 ± 0.8	275.5 ± 26.0	3626 ± 407
CA1	SAL	6.5 ± 0.3	14.1 ± 0.4	3.1 ± 0.2	118.3 ± 6.3	1942 ± 86
	LPS	7.0 ± 0.4	14.7 ± 0.4	3.3 ± 0.3	126.5 ± 12.0	1741 ± 240
CA3	SAL	6.5 ± 0.3	14.3 ± 0.3	1.5 ± 0.1	61.0 ± 2.3	1014 ± 50
	LPS	7.0 ± 0.4	13.7 ± 0.5	1.6 ± 0.2	65.0 ± 6.1	930 ± 125
DG	SAL	6.3 ± 0.3	14.1 ± 0.4	1.9 ± 0.1	118.3 ± 6.3	1030 ± 77
	LPS	7.0 ± 0.4	14.7 ± 0.4	2.1 ± 0.3	126.5 ± 12.0	955 ± 94

Table S1. Stereological parameters

Values are means ± SEM (N = 4 per group). A Mann and Whitney rank sum test was used.
