# Supplementary material for: Tiagabine Improves Hippocampal Long-Term Depression in Rat Pups Subjected to Prenatal Inflammation
Source: PLoS One. 2014 Sep 3;9(9):e106302. doi: 10.1371/journal.pone.0106302 (PMC4153642; doi:10.1371/journal.pone.0106302)
Supplement: Methods S1 — Specific methods used for obtaining supporting information. qRT-PCR. Paired-pulse depression analysis. (DOCX) [file pone.0106302.s006.docx]

**Methods S1**

## qRT-PCR

Following decapitation, the brain from each rat was quickly dissected on ice. The right and left hippocampus were extracted, immediately immersed in liquid nitrogen and conserved at -80°C until further processing. Tissue lysis and homogenization was performed with a rotor-stator. Total RNA was extracted employing the RNeasy® Plus Kit (Qiagen; Valencia, CA, USA). RNA concentration and purity were verified by the measurement of the 260 and 280 nm absorbances with a NanoDrop™8000 spectrophotometer. RNA integrity was assessed with a 2100 Bioanalyzer (Agilent; Santa Clara, CA, USA). Reverse transcription was then performed using Superscript II reverse transcriptase (Invitrogen) and random hexamers following the manufacturer’s instructions. PCR primers were designed with online-available Primer 3 Plus software, in order to cover inter-exon junctions and avoid genomic DNA amplification. The following primers (5’ to 3’) were used: actin (forward, 5’-GGCATTGTCACCAACTGGG-3’; reverse, 5’-CATACAGGGACAACACAGCC-3’), GAD65 (forward :5’-TATGACACGGGAGACAAGGC-3’; reverse : 5’-AGACATTTGTGTGCTGAGGC-3’), GAD76 (forward : 5’-GTTTTGGACTTCCACCACCC-3’; reverse : 5’-AGTTGATGTCAGCCATTCGC-3’), GAT1 (forward, 5’-TCATCTTCTCCATCGTGGGC-3’; reverse, 5’-CAGAACTGGCTGTCAATGCC-3’), VGAT (forward, 5’-AGATCCTCATCGCGTGCC-3’; reverse, 5’-AAACTGTTGTACATGAGGTTGCC-3'), α2 GABA_A_-receptor subunit (forward, 5’CTCCCAAGTGTCATTCTGGC-3’; reverse, 5’-AAGGCAGAGAACACAAACGC-3’), α3 GABA_A_-receptor subunit (forward, 5’- TGTCATCCAGACCTACTTGCC-3’; reverse, 5’-ACAAAGGCATAACAGACGGC-3’), α5 GABA_A_-receptor subunit (forward, 5’-ATGCAGCTTGAGGACTTCCC-3’; reverse, 5’-GTGCTGATGTTCTCAGTGCC-3’). Equal amounts of cDNA were analyzed in triplicate for each primer pair. PCR was performed with the Light Cycler 480 (Roche) with a denaturing temperature of 95°C, a melting temperature of 63°C, and an elongation temperature of 72°C. The expression of target genes was normalized to the expression of β actin.

## Paired-pulse depression analysis

The paired-pulse depression (PPD) of eIPSCs was obtained by applying pairs of identical shocks delivered with increasing interval time durations of 100, 200, 300 and 400 ms. PPD was estimated by calculating the “PPD ratio” by normalizing the second eIPSC amplitude to the first one. The contribution of presynaptic GABA_B_ receptor to PPD was tested by applying a GABA_B_ receptor antagonist (CGP 55845; Tocris Bioscience). PPD profiles obtained with increasing inter-pulse intervals were compared between SAL and LPS animals, in either the absence or presence of 1 μM CGP 55845, to determine the impact of prenatal LPS on the activity of presynaptic GABA_B_ receptors.
